# Effects of H_2_ High-pressure Annealing on HfO_2_/Al_2_O_3_/In_0.53_Ga_0.47_As Capacitors: Chemical Composition and Electrical Characteristics

**DOI:** 10.1038/s41598-017-09888-6

**Published:** 2017-08-29

**Authors:** Sungho Choi, Youngseo An, Changmin Lee, Jeongkeun Song, Manh-Cuong Nguyen, Young-Chul Byun, Rino Choi, Paul C. McIntyre, Hyoungsub Kim

**Affiliations:** 1 School of Advanced Materials Science and Engineering, Sungkyunkwan University, Suwon, 16419 Republic of Korea; 2 Department of Materials Science and Engineering, Inha University, Incheon, 22212 Republic of Korea; 3 ASM International, Phoenix, AZ 85034 USA; 4 Department of Materials Science and Engineering, Stanford University, Stanford, CA 94305 USA; 5 SKKU Advanced Institute of Nanotechnology (SAINT), Sungkyunkwan University, Suwon, 16419 Republic of Korea

## Abstract

We studied the impact of H_2_ pressure during post-metallization annealing on the chemical composition of a HfO_2_/Al_2_O_3_ gate stack on a HCl wet-cleaned In_0.53_Ga_0.47_As substrate by comparing the forming gas annealing (at atmospheric pressure with a H_2_ partial pressure of 0.04 bar) and H_2_ high-pressure annealing (H_2_-HPA at 30 bar) methods. In addition, the effectiveness of H_2_-HPA on the passivation of the interface states was compared for both p- and n-type In_0.53_Ga_0.47_As substrates. The decomposition of the interface oxide and the subsequent out-diffusion of In and Ga atoms toward the high-*k* film became more significant with increasing H_2_ pressure. Moreover, the increase in the H_2_ pressure significantly improved the capacitance‒voltage characteristics, and its effect was more pronounced on the p-type In_0.53_Ga_0.47_As substrate. However, the H_2_-HPA induced an increase in the leakage current, probably because of the out-diffusion and incorporation of In/Ga atoms within the high-*k* stack.

## Introduction

For high-speed metal-oxide-semiconductor field-effect transistors (MOSFETs) with a technology node of less than 5 nm, In_1−*x*_Ga_*x*_As has been considered the most promising channel layer as a replacement for conventional Si because of its various merits such as high electron mobility, large band gap, and small lattice mismatch with InP (for practical device integration on a Si wafer)^1–3^. Therefore, tremendous efforts have been made to engineer the atomic layer deposition (ALD) of high-*k* dielectrics on In_1−*x*_Ga_*x*_As and to improve the electrical properties of MOS capacitors using various pre- and/or post-deposition processes.

Since the use of conventional Si-channel MOSFET devices, post-metallization annealing (PMA), also termed as forming gas annealing (FGA) and typically performed at 300‒400 °C in a N_2_ atmosphere mixed with a small amount of H_2_, has been the most effective method for passivating the interface states (specifically, the dangling bonds) located within a Si band gap^4–6^. Similarly, a decrease in the interface state density (*D*_it_) has been achieved for MOS capacitors made of high-*k* dielectrics deposited on III-V channel materials, including In_1−*x*_Ga_*x*_As, by FGA^7–9^. In addition, a further reduction in *D*_it_ could be achieved for high-*k*/n-type In_1−*x*_Ga_*x*_As MOS capacitors using H_2_ high-pressure annealing (H_2_-HPA)^10^, whose effectiveness has been demonstrated in a high-*k*/Si system^11–13^. However, its effectiveness on In_1−*x*_Ga_*x*_As substrates with different doping types was not compared in detail.

Many studies have experimentally evidenced the adverse effects of PMA such as substantial out-diffusion of components from the III-V interface oxide toward the high-*k* film in various high-*k*/III-V MOS capacitors^14–20^. For instance, Krylov *et al*.^14, 15^ observed leakage current degradation of Al_2_O_3_ on In_0.53_Ga_0.47_As after N_2_ annealing/FGA at 400 °C and attributed it to significant In out-diffusion. The out-diffusion of Ga and As was also noted at higher temperatures of 400−700 °C under N_2_ atmosphere^16^. For HfO_2_ dielectrics on In_1−*x*_Ga_*x*_As, both In and Ga out-diffusion occurred at temperatures higher than 400 °C^17^, and In desorption/migration was enhanced by FGA at temperatures as low as 350 °C^18^.

Herein, we studied the effect of H_2_ pressure on the chemical/electrical properties of HfO_2_/Al_2_O_3_ gate dielectrics deposited *via* ALD on HCl wet-cleaned In_0.53_Ga_0.47_As substrates. Possible out-diffusion of the substrate elements and their subsequent incorporation into the high-*k* film were examined with different H_2_ pressures using conventional FGA (at atmospheric pressure using 4% H_2_ in N_2_) and H_2_-HPA (at 30 bar using 100% H_2_) methods. Furthermore, for a detailed study of their respective effects on the electrical characteristics of MOS capacitors, both p- and n-type In_0.53_Ga_0.47_As substrates were used.

## Results and Discussion

### Chemical Composition

**Figure 1:**
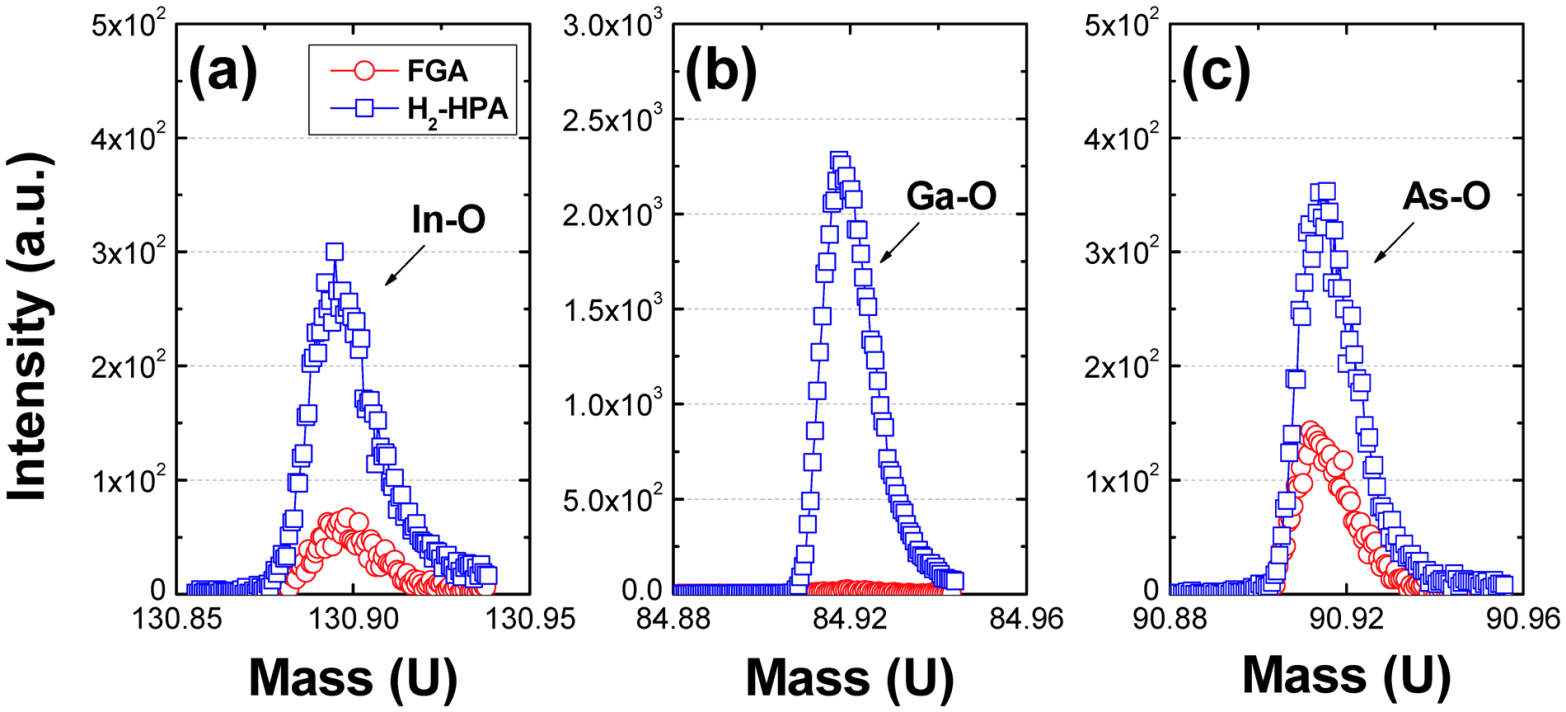
Changes in the ToF-SIMS intensities of (**a**) In, (**b**) Ga, and (**c**) As collected from the surface of the HfO_2_/Al_2_O_3_/In_0.53_Ga_0.47_ As samples with different PMA conditions.

**Figure 2:**
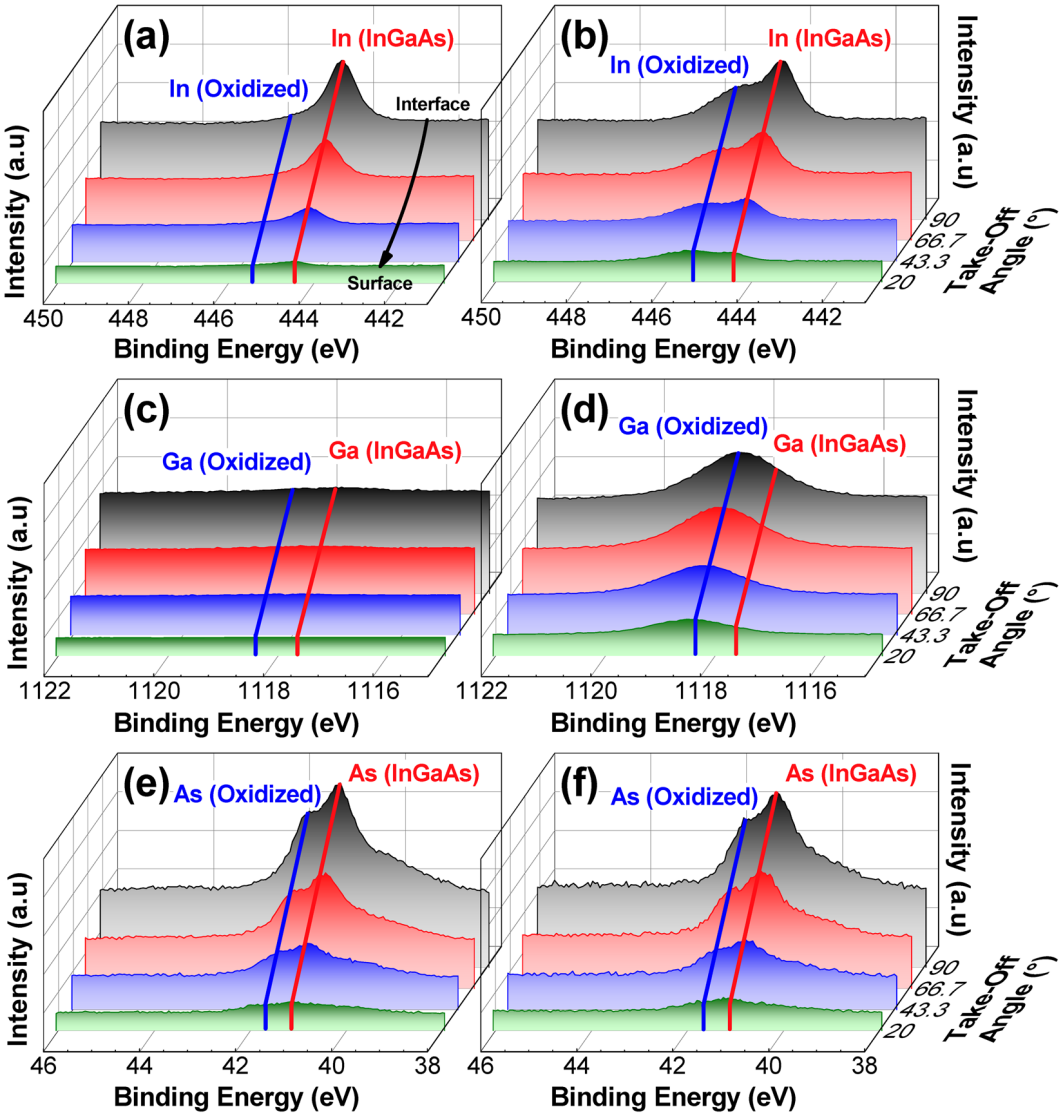
Angle-resolved XPS spectra of (**a**,**b**) In 3*d*, (**c**,**d**) Ga 2*p*, and (**e**,**f**) As 3*d* peaks measured from the HfO_2_/Al_2_O_3_ films on In_0.53_Ga_0.47_As after PMA at 400 °C for 30 min: (**a**,**c**,**e**) FGA and (**b**,**d**,**f**) H_2_-HPA.

First, the compositional changes in the HfO_2_/Al_2_O_3_ gate dielectric due to different PMA conditions (FGA at atmospheric pressure using 4% H_2_ gas balanced with N_2_ and H_2_-HPA at 30 bar using 100% H_2_ gas) were examined using time-of-flight secondary ion mass spectrometry (ToF-SIMS) and angle-resolved X-ray photoelectron spectroscopy (ARXPS), and the results are shown in Figs 1 and 2, respectively. A thin dielectric stack of 2 nm HfO_2_/1 nm Al_2_O_3_ was intentionally used because of the short information depth of XPS measurements, whereas ToF-SIMS was used to detect the chemical change on the high-*k* surface to a depth of couple of angstroms. Although the thinness of the gate dielectric stack and the PMA performed in the absence of a gate metal electrode might yield results different from those of the thicker dielectrics used for electrical characterization, it is believed that a relative comparison would be possible between the samples with different PMA conditions. According to the ToF-SIMS data shown in Fig. 1, some amounts of substrate elements, mainly In and As, were detected on the sample surface after FGA, which indicated that the out-diffusion of the substrate elements occurred during the ALD^21^ and/or the subsequent FGA process^9, 14, 18, 19, 22^. When the H_2_ pressure was increased to 30 bar, the concentrations of In, Ga, and As atoms on the high-*k* surface increased significantly as compared to those in the FGA (H_2_ partial pressure of 0.04 bar) sample. This suggests that the H_2_ pressure strongly affects the out-diffusion of the substrate elements. This was further confirmed by ARXPS measurements, as shown in Fig. 2; additional comparison with the H_2_-HPA sample annealed at a different H_2_ pressure of 10 bar can be found in Figure S1 (Supplementary Information). As the H_2_ pressure was increased, both In and Ga atoms (in their oxidized states) significantly diffused toward the dielectric surface; this verified the ToF-SIMS result. In contrast, no significant change was observed in the intensity of the As-O peaks in the depth direction of the As 3d spectrum for different PMA conditions. Because the chemical information of the bulk region above the interface was also gathered at the highest take-off angle of 90°, it was difficult to differentiate the possible change in the amount of interfacial oxide while varying the H_2_ pressure.

The In_0.53_Ga_0.47_As substrate was exposed to air after the removal of native oxide by wet chemical cleaning, and the subsequent ALD high-*k* process was carried out in a highly oxidizing atmosphere at an elevated temperature. Therefore, it is most likely that an abrupt interface between the high-*k* and In_0.53_Ga_0.47_As was not realized^23^, and the formation of In- and Ga-oxides was thermodynamically preferred to that of As-oxide^24^. As suggested by several researchers^24–26^, the formed interfacial In- and Ga-oxides can be decomposed by atomic hydrogen at temperatures as low as 400 °C. Therefore, a high H_2_ pressure could accelerate their decomposition and the subsequent release of In/Ga-related species into the high-*k* film. Similar to this result, Cabrera *et al*.^18^ observed an enhanced decomposition of the interface oxide and the subsequent out-diffusion of In atoms in a HfO_2_/In_0.53_Ga_0.47_As system when the PMA ambient (at atmospheric pressure) was changed from pure N_2_ to 5% H_2_ at 350 °C. However, they did not observe noticeable Ga out-diffusion^18^. The contradictory result might be because of differences in the FGA temperature, the use of characterization tools with different detection limits, and the H_2_ pressure. On the other hand, As atoms preferred to diffuse out fast in a gaseous state at low temperatures with some amount of aggregation at the high-*k*/III-V interfaces^20^. Therefore, the increase in the H_2_ pressure may not significantly affect its distribution within the bulk region of the high-*k* gate stack, as observed from the ARXPS measurement (Fig. 2). Instead, a slight enrichment of As atoms on the high-*k* surface occurred, as observed from the ToF-SIMS measurement (Fig. 1). Because the out-diffused In/Ga-related species might exist in an oxidized form and produce point defects in the high-*k* film, they may generate additional trap energy levels in the band gap of the high-*k* film, which might degrade the electrical properties of the high-*k* film itself, such as the leakage current and reliability, rather than increasing *D*_it_.

### Electrical Characteristics

**Figure 3:**
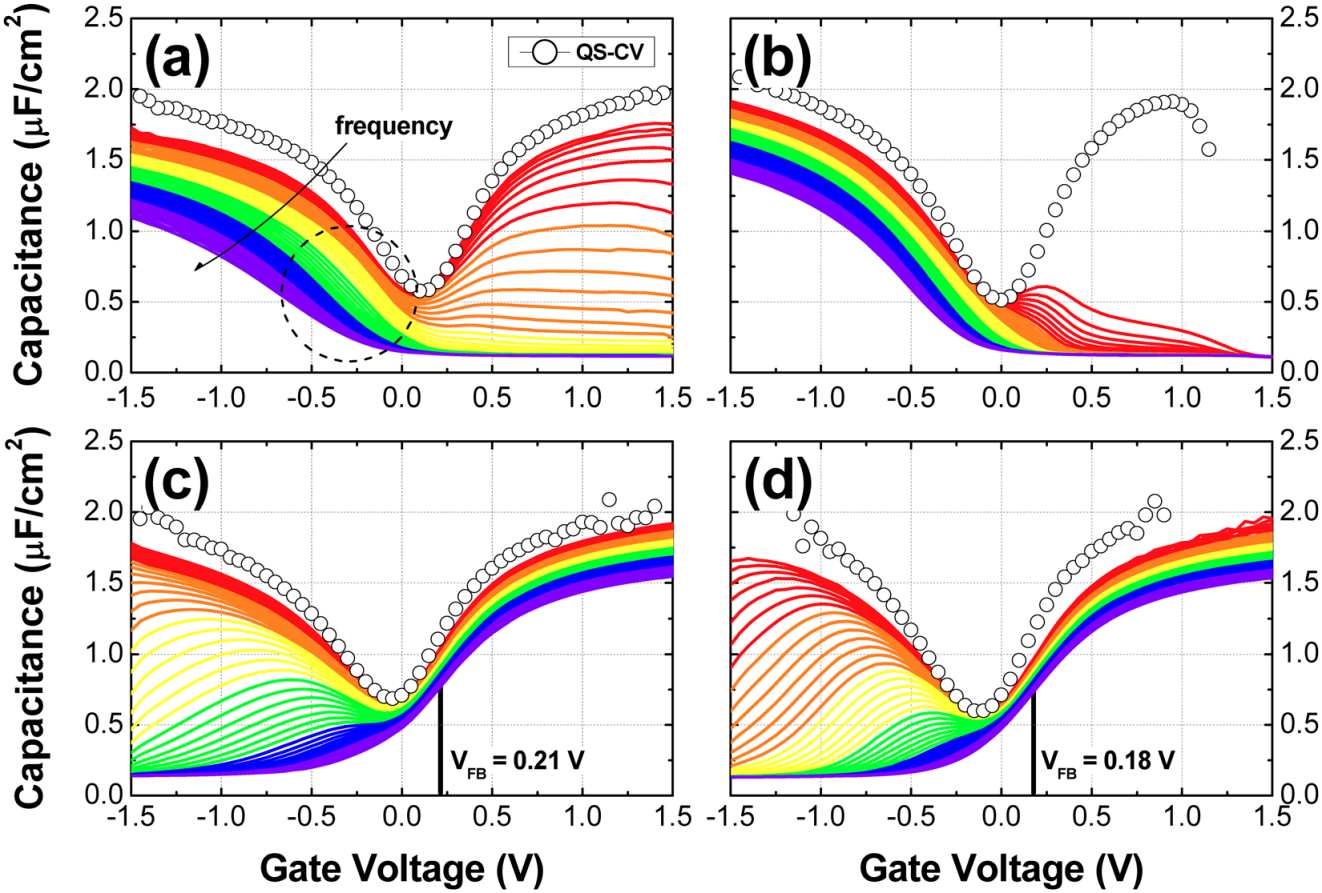
Quasi-static and high-frequency (100 Hz‒1 MHz) *C*–*V* characteristics of the HfO_2_/Al_2_O_3_ dielectrics on (**a**,**b**) p-type and (**c**,**d**) n-type In_0.53_Ga_0.47_As substrates after PMA at 400 °C for 30 min: (**a**,**c**) FGA and (**b**,**d**) H_2_-HPA.

For electrical characterization of the HfO_2_/Al_2_O_3_ dielectrics on both p- and n-type In_0.53_Ga_0.47_As after PMA at different H_2_ pressures, HfO_2_ and Al_2_O_3_ layers with thicknesses of approximately 4.5 and 1.0 nm, respectively, were deposited and MOS capacitors were fabricated. Figure 3 shows the capacitance−voltage (*C*–*V*) characteristics of the MOS capacitors fabricated on the p- and n-type In_0.53_Ga_0.47_As substrates after PMA at different H_2_ pressures. In addition to quasi-static (QS) *C*–*V* measurements, a series of high-frequency *C*–*V* measurements were carried out at frequencies varying from 100 Hz to 1 MHz. The flat band voltage (*V*_FB_) was extracted using the inflection point method^27, 28^ and was included in the *C*–*V* graphs of the n-type In_0.53_Ga_0.47_As samples shown in Fig. 3. For the p-type In_0.53_Ga_0.47_As samples, it was difficult to determine the accurate *V*_FB_ values due to a significantly large frequency dispersion at the flat band condition. In the case of the reference samples (FGA samples), a large frequency-dependent hump from depletion to inversion regions was observed on both p- and n-type In_0.53_Ga_0.47_As substrates (Fig. 3a and c), which indicates the existence of a high density of interface states and the occurrence of strong Fermi-level pinning^8, 29^. For the p-type In_0.53_Ga_0.47_As substrate after FGA, a large amount of dispersion at the accumulation region (at a negative bias) was observed. This abnormal *C*‒*V* behavior can be attributed to border traps^7^ (or disorder-induced gap states^30^) in the defective near-interface region, probably originating from the wet-chemical cleaning, *ex situ* ALD process, damage that occurred during the electrode deposition, etc^22^. In addition, the comparison with the n-type In_0.53_Ga_0.47_As sample (Fig. 3c) indicates that a larger number of border traps existed near the valence band (VB) edge of In_0.53_Ga_0.47_As than that near the conduction band (CB) edge of In_0.53_Ga_0.47_As. However, considering the largest dispersion and stretch-out of the frequency-dependent *C*‒*V* curves near *V*_FB_ (see the black dashed circle in Fig. 3a), there is also a possibility of an additional strong Fermi-level pinning effect due to the high density of interface states located close to the VB edge of the In_0.53_Ga_0.47_As band gap. In addition, a systematic increase in the frequency-dependent hump at a gate bias range of −1.5 V to 0 V for the FGA sample on the n-type In_0.53_Ga_0.47_As suggests strong Fermi-level pinning by the high density of interface states. When H_2_-HPA was performed, the observed hump and the stretch-out of the *C*–*V* curve were significantly suppressed on both p- and n-type In_0.53_Ga_0.47_As substrates (see Fig. 3b and d). This improvement suggests effective passivation of the interface states (probably the dangling bonds of the substrate elements) by hydrogen^7–9^. In addition to the passivation of interface traps, most recently, Tang *et al*. reported a simultaneous reduction in the border trap density for Al_2_O_3_/n-In_0.53_Ga_0.47_As capacitors as a result of FGA^9^. However, this effect was not clearly noticeable in the case of the n-type In_0.53_Ga_0.47_As substrate when H_2_-HPA was performed, probably due to the larger number of border traps (related to the degree of frequency dispersion in accumulation^7^) created by the existence of a highly defective near-interface region originating from different sample preparation conditions.

**Figure 4:**
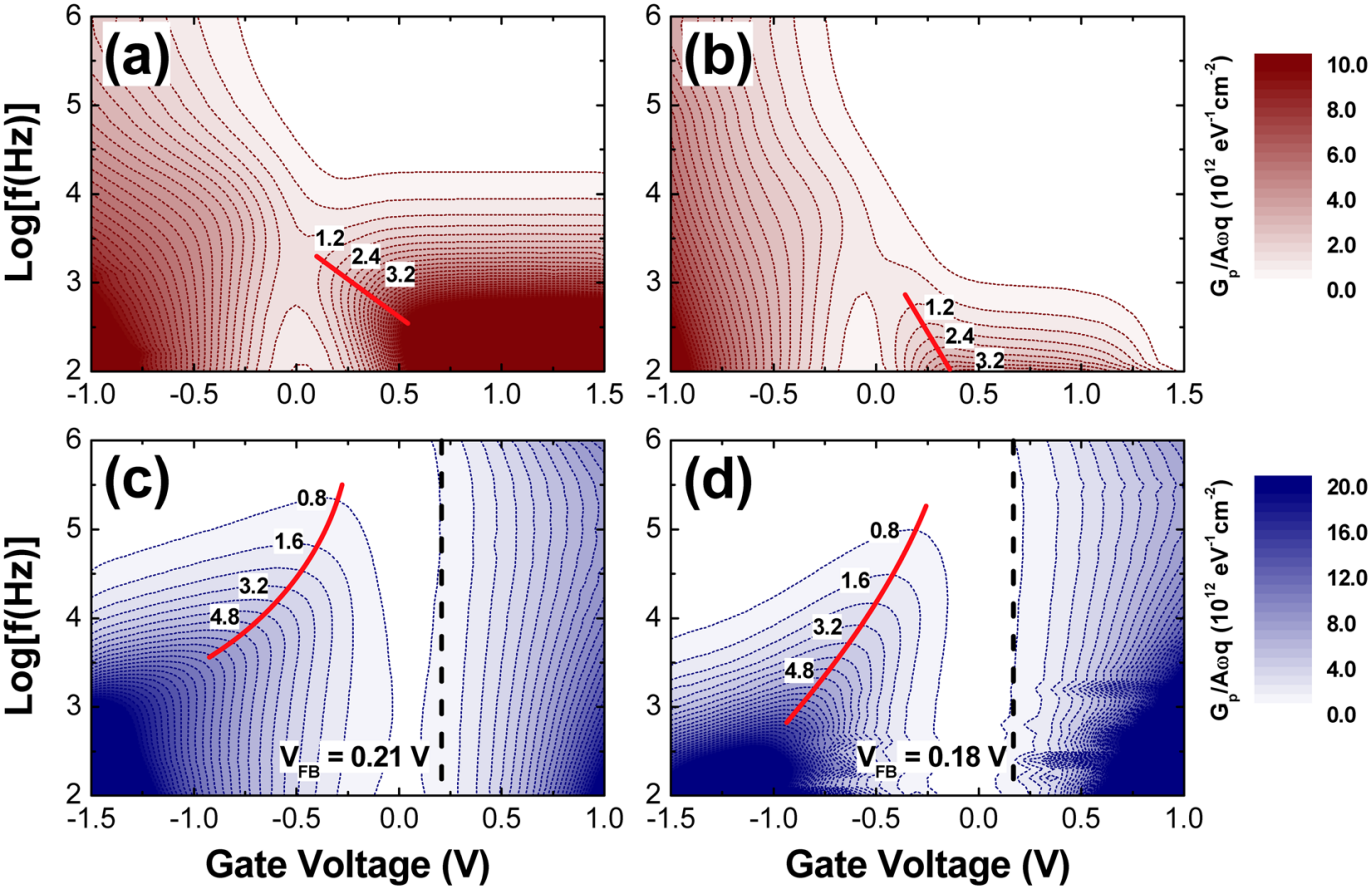
Normalized parallel conductance (*G*_p_/*ωqA*) as a function of the gate voltage and frequency measured from (**a**,**b**) p-type and (**c**,**d**) n-type In_0.53_Ga_0.47_As MOS capacitors. Here, *ω* is the measurement angular frequency and *A* is the capacitor area. The samples were subjected to PMA at 400 °C for 30 min: (**a**,**c**) FGA and (**b**,**d**) H_2_-HPA.

To closely observe the effect of H_2_-HPA on the passivation of the interface states, we examined the degree of Fermi-level movement by drawing two-dimensional contour plots of parallel conductance (*G*_p_)^4, 29^, as given in Fig. 4. *G*_p_ was determined from the following equation:

1 $${G}_{p}=\frac{{\omega }^{2}{C}_{ox}^{2}{G}_{m}}{{G}_{m}^{2}+{\omega }^{2}{({C}_{ox}-{C}_{m})}^{2}},$$

where *C*_*m*_ and *G*_*m*_ are the measured capacitance and conductance, respectively, at different frequencies in the parallel mode, *ω* is the angular frequency, and *C*_*ox*_ is the oxide capacitance^4, 29^. The *C*_*ox*_ value was assumed to be the accumulation capacitance determined from the QS *C*–*V* curve. The solid trace lines of the peak *G*_p_ values in Fig. 4 are indicative of the degree of Fermi-level pinning^8, 29^. When compared to the FGA sample, the H_2_-HPA samples on both p- and n-type In_0.53_Ga_0.47_As layers exhibited trace lines with a steeper slope with varying gate voltages, which indicated more alleviated Fermi-level pinning and coincided well with the frequency-dependent *C*–*V* behavior shown in Fig. 3. In addition, considering the *C*–*V* analysis results shown in Figs 3 and 4, one of the most notable results is the much greater effectiveness of H_2_-HPA in suppressing *D*_it_ in the top half of the In_0.53_Ga_0.47_As band gap. This is apparent in the much smaller dispersive feature in inversion for the p-type sample (comparing Fig. 3b with Fig. 3a) and in the much steeper trajectory of the normalized *G*_p_ (comparing Fig. 4b with Fig. 4a). The improvement in the interface trap response in the bottom half of the In_0.53_Ga_0.47_As band gap (n-type substrates measured in inversion) for the H_2_-HPA sample is less obvious than in the case of the reference FGA process. Therefore, it seems that H_2_-HPA is good for repairing/passivating the interface traps between the midgap and the CB edge but not for those near the VB edge.

These results suggest that annealing under high-pressure H_2_ reduces the density of Ga dangling bonds or anti-site defects at the interface because these defects have transition-state energies centered near the CB edge of GaAs^31^ and may tail into the upper half of the In_1−*x*_Ga_*x*_As band gap. Apparently, the annealing is less effective in removing As dangling bonds, which have an energy level near the VB edge^31^. Although the exact reason why it happens this way is not clear yet, there is a possibility that the atomic hydrogen will bind more strongly to group III atoms at the interface, considering that the Pauling electronegativity differences for In‒H and Ga‒H are larger than those for As‒H^32^. Meanwhile, because In and Ga seem to diffuse into the high-*k* film much more readily under increased H_2_ pressure, this would tend to generate As dangling bonds at the interface region. Therefore, it is possible that more As dangling bonds are passivated by the H_2_-HPA, but more are generated at the same time by In and Ga out-diffusion. Because we do not have a clear picture of exactly which of these two effects (hydrogen passivation versus new dangling bond generation) is most important, these hypotheses should be tested in our future work.

**Figure 5:**
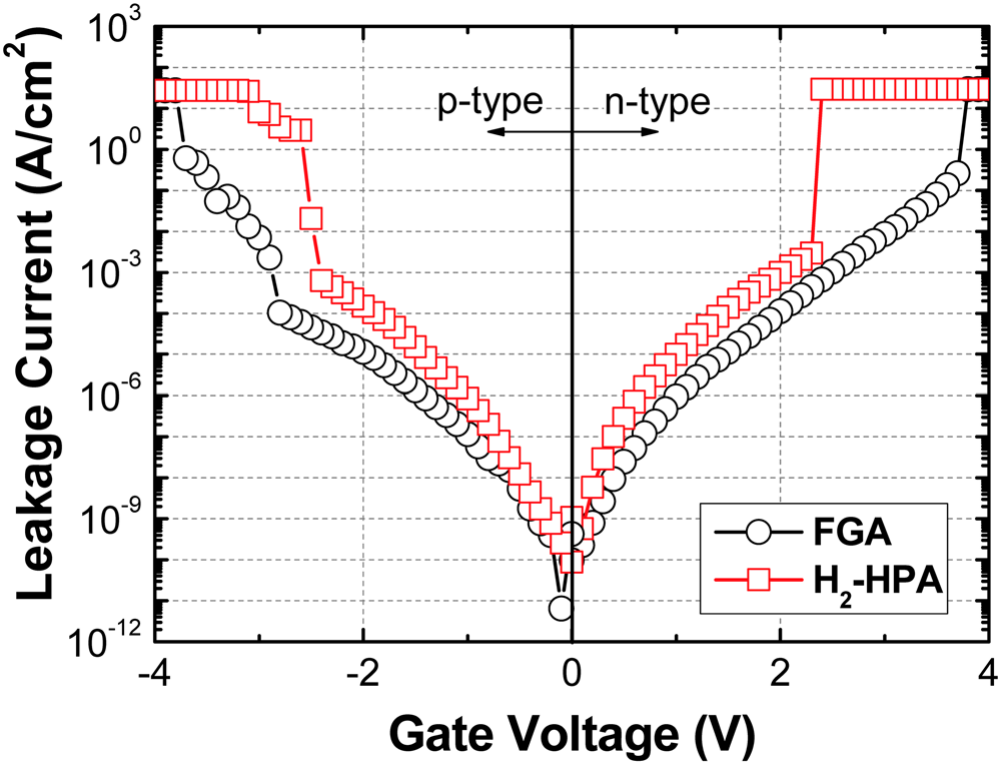
Leakage current density vs. gate voltage for HfO_2_/Al_2_O_3_ dielectrics on p- and n-type In_0.53_Ga_0.47_As substrates after FGA and H_2_-HPA.

Figure 5 shows the leakage current characteristics of the MOS capacitors on both p- and n-type In_0.53_Ga_0.47_As substrates, where the gate bias was applied under an electron injection condition from the gate and substrate sides, respectively. Regardless of the substrate doping type, the leakage current increased by approximately one order of magnitude at ±2.0 V when the H_2_ pressure was increased from 0.04 to 30 bar. As evidenced from the chemical analyses results, the incorporation of more In‒O/Ga‒O bonds and the resulting formation of trap states within the high-*k* film by the high H_2_ pressure may be plausible explanations for the degraded leakage current characteristics.

## Conclusion

In summary, we investigated the effects of H_2_-HPA on the out-diffusion of substrate elements and the electrical properties of an ALD-HfO_2_/Al_2_O_3_ gate stack on In_0.53_Ga_0.47_As with different doping types. As the H_2_ pressure was increased from 0.04 (FGA) to 30 bar (H_2_-HPA) under an identical thermal budget (400 °C for 30 min), the out-diffusion of In and Ga elements into the high-*k* dielectric stack was significantly enhanced. In comparison to conventional FGA, H_2_-HPA significantly alleviated the Fermi-level pinning of the HfO_2_/Al_2_O_3_/In_0.53_Ga_0.47_As MOS capacitors by passivating the interface states; this effect was more pronounced on the p-type In_0.53_Ga_0.47_As than on the n-type In_0.53_Ga_0.47_As. However, when H_2_-HPA was used, the leakage current characteristics were somewhat degraded on both p- and n-type In_0.53_Ga_0.47_As substrates. This is believed to be affected by the enhanced In/Ga incorporation and the subsequent defect formation within the high-*k* stack because of the high H_2_ pressure. As a result, in the future, optimization of the H_2_ pressure will be needed to minimize the degradation of the leakage current characteristics while achieving improved interface properties with In_0.53_Ga_0.47_As substrates.

## Methods

### MOS Capacitor Fabrication

MOS capacitors were fabricated on both p- and n-type In_0.53_Ga_0.47_As layers epitaxially grown on InP. The In_0.53_Ga_0.47_As/InP wafers were supplied by Intelligent Epitaxy Technology, Inc., and their specification is provided in Table 1. Before the high-*k* gate dielectric deposition, the cleaved substrates were cleaned using a 10% HCl aqueous solution for 30 s. A stacked high-*k* dielectric of 4.5 nm HfO_2_/1.0 nm Al_2_O_3_ was deposited *in situ* using trimethylaluminum (TMA)−H_2_O and tetrakis(ethylmethylamino)hafnium (TEMAHf)−H_2_O precursor combinations at 200 °C. The prepared films followed a gate metallization step, *i.e*., a lift-off process using a sputter-deposited TaN (50 nm) electrode with a Ni (10 nm) capping layer. Afterwards, conventional FGA and H_2_-HPA were performed at 400 °C for 30 min prior to the electrical characterization. The FGA was performed at atmospheric pressure using 4% H_2_ gas balanced with N_2_, which corresponds to a H_2_ partial pressure of 0.04 bar. For H_2_-HPA, 100% H_2_ was used at a high pressure of 30 bar.

**Table 1:** Specifications of the In_0.53_Ga_0.47_As/InP wafers used in this experiment.

Substrates	Layers	Thickness (nm)	*x*	Dopant	Doping conc.(cm^−3^)
p-type In_1−*x*_Ga_*x*_As on p^+^ InP	In_1−*x*_ Ga_*x*_As	100	0.53	Be	5 × 10^17^
	In_1−*x*_Ga_*x*_As	150	0.53	Be	1 × 10^17^
	p^+^ InP	650,000	—	Zn	2 × 10^18^
n-type In_1−*x*_Ga_*x*_As on n^+^ InP	In_1−*x*_Ga_*x*_As	150	0.53	Si	5 × 10^17^
	In_1−*x*_Ga_*x*_As	100	0.53	Si	1 × 10^17^
	n^+^ InP	650,000	—	S	3 × 10^18^

### Measurement and Characterization

For the electrical characterization of the fabricated MOS capacitors, an Agilent E4980A LCR meter, an Agilent B1500A semiconductor device analyzer, and Keithley 6514 electrometer/230 programmable voltage source were used. In addition, the compositional change in the high-*k* films induced by different PMA conditions were probed by ToF-SIMS (TOF-SIMS 5, ION-TOF), and ARXPS (K-alpha, Thermo Scientific Inc.) with an Al K_α_ (1486.6 eV) source. For the ARXPS measurement, the take-off angle was varied from 20° to 90°, and a pass energy of 20 eV was used.

## Electronic supplementary material

Supplementary Information
