# Supplementary material for: Effects of H2 High-pressure Annealing on HfO2/Al2O3/In0.53Ga0.47As Capacitors: Chemical Composition and Electrical Characteristics
Source: Sci Rep. 2017 Aug 29;7:9769. doi: 10.1038/s41598-017-09888-6 (PMC5575061; doi:10.1038/s41598-017-09888-6)
Supplement: Supplementary file 1 — Supplementary Information [file 41598_2017_9888_MOESM1_ESM.pdf]

Supplementary Information

Effects of H_2 High-pressure Annealing on $HfO_2/Al_2O_3/In_{0.53}Ga_{0.47}As$ Capacitors: Chemical Composition and Electrical Characteristics

Sungho Choi[1], Youngseo An[1], Changmin Lee[1], Jeongkeun Song[1], Manh-Cuong Nguyen[2], Young-Chul Byun[3], Rino Choi[2], Paul C. McIntyre[4] & Hyoungsub Kim[1,5*]

[1] School of Advanced Materials Science and Engineering, Sungkyunkwan University, Suwon 16419, Republic of Korea

[2] Department of Materials Science and Engineering, Inha University, Incheon 22212, Republic of Korea

[3] ASM International, Phoenix, AZ 85034, USA

[4] Department of Materials Science and Engineering, Stanford University, Stanford, CA 94305, USA

[5] SKKU Advanced Institute of Nanotechnology (SAINT), Sungkyunkwan University, Suwon, 16419, Republic of Korea

Correspondence and requests for materials should be addressed to H. Kim (email: hsubkim@skku.edu)

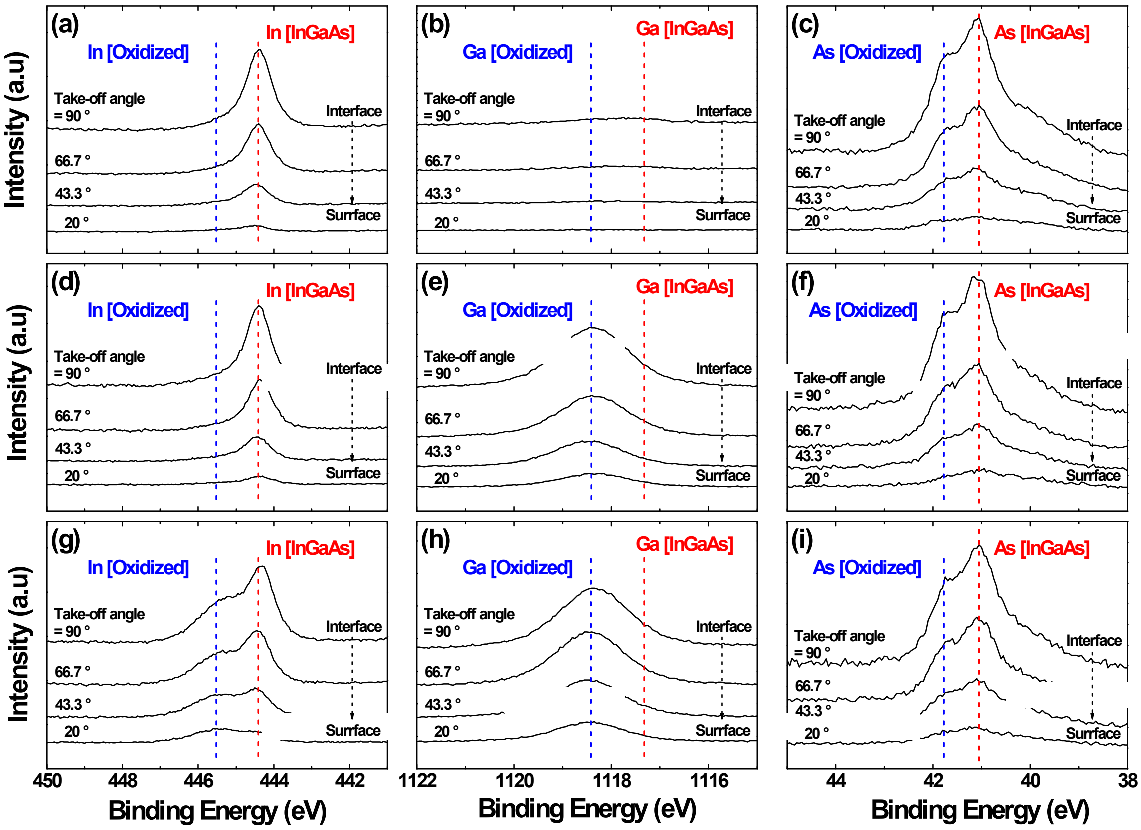

Figure S1. Angle-resolved XPS spectra of (a, d, g) In 3*d*, (b, e, h) Ga 2*p*, and (c, f, i) As 3*d* peaks measured from the HfO_2/Al_2O_3 films on $In_{0.53}Ga_{0.47}As$ films after different PMA at 400 °C for 30 min: (a, b, c) FGA, (d, e, f) H_2-HPA at 10 bar, and (g, h, i) H_2-HPA at 30 bar.
